# NKCC1 promotes proliferation, invasion and migration in human gastric cancer cells via activation of the MAPK-JNK/EMT signaling pathway

**DOI:** 10.7150/jca.49709

**Published:** 2021-01-01

**Authors:** Jun-fu Wang, Kun Zhao, Ye-yang Chen, Yue Qiu, Jin-hui Zhu, Bo-pei Li, Zheng Wang, Jun-qiang Chen

**Affiliations:** Department of Gastrointestinal Surgery, The First Affiliated Hospital of Guangxi Medical University, 6 Shuangyong Road, Nanning 530021, Guangxi Zhuang Autonomous Region, China.

**Keywords:** Na+/K+/2CI- cotransporter 1 (NKCC1), gastric cancer, migration, invasion, epithelial-mesenchymal transition

## Abstract

**Aims:** This study aimed to explore the function of NKCC1 in the proliferation, migration and invasion of Gastric cancer (GC) cells.

**Materials and Methods:** GC data extracted from the database was analyzed using molecular bioinformatics. The expression levels of NKCC1 in tissue samples from GC patients and GC cell lines were determined by Western blotting, qRT-PCR, and immunohistochemistry. Immunofluorescence was used to detect protein localization. The GC cell lines were transfected with NKCC1-shRNA or expression plasmid, and *in vitro* proliferation, invasion and migration were analyzed by the CCK8, wound healing and transwell tests.

**Results:** The NKCC1 mRNA level was significantly increased in GC tissues than that in normal gastric tissues (*P* = 0.0195). This phenomenon was further confirmed by the analysis of the TCGA-GTEx database that includes 408 gastric cancer tissues and 211 normal gastric tissues (*P* < 0.01). Furthermore, the increased level of NKCC1 was significantly correlated with Tumor size (*P* = 0.039), lymphatic node metastasis (*P* = 0.035) and tumor stage (*P* = 0.034). *In vitro* experiments confirmed that NKCC1 expression was higher in GC cells compared to that in GES-1 cells, and was mainly localized to the cytoplasm and membrane. NKCC1 silencing inhibited GC cell proliferation, invasion, migration and EMT, whereas its overexpression had the opposite effects. Furthermore, NKCC1 overexpression upregulated and activated JNK, and the targeted inhibition of JNK by SP600125 abrogated the pro-metastatic effects of NKCC1.

**Conclusions:** NKCC1 promotes migration and invasion of GC cells by MAPK-JNK/EMT pathway and can be a potential therapeutic target.

## Introduction

Gastric cancer (GC) originates from the gastric epithelial cells, and is one of the most commonly diagnosed malignancies and the third leading cause of cancer-related deaths worldwide 1-3. The occurrence and development of GC are controlled by multiple genes and factors. Furthermore, due to lack of sensitive non-invasive diagnostic indicators, most patients are diagnosed in the advanced stages. In addition, the post-surgery five-year survival rate of gastric cancer is only 20% 4 owing to the high rates of local and distal metastasis and postoperative recurrence 5-6. Therefore, it is vital to elucidate the mechanisms underlying GC metastasis in order to identify novel early diagnostic markers and therapeutic targets, and improve patient prognosis.

Na+/K+/2CI- cotransporter 1 (NKCC1) is a member of the SLC12 family of cationic chloride co-transporter membrane proteins, including NKCC1 and NKCC2 isotypes 7-8. NKCC1 transports Na, K, and Cl ions across epithelial and non-epithelial cells, thereby regulating cell proliferation, differentiation and metastasis 9-11. It is present in the stomach, esophagus, colorectal, liver, pancreas, lung, and other tissues and is aberrantly expressed in many tumors. High levels of NKCC1 in glioma tissues affect the shape, polarity, and adhesion of tumor cells 10. In addition, NKCC1 promotes the growth of GC cells 9, although its potential role in GC metastasis has not been elucidated.

Epithelial-mesenchymal transition (EMT) refers to the transformation of polar epithelial cells into metastatic mesenchymal cells. This process endows tumor cells with the ability to metastasize and invade distant tissues 12. It is initiated with the degradation or redistribution of connexins and dissociation of the β-catenin/E-cadherin complex, resulting in the loss of cell stability, polarity and intercellular adhesion, and cytoskeletal rearrangement from keratin to vimentin 13-15. The tumor cells acquire characteristics of embryonic mesenchymal cells and become more invasive following EMT, resulting in greater infiltration into adjacent tissues, blood, or lymphatic vessels and eventually distant metastasis 16-18. EMT is regulated by multiple pathways, including NF-kB, Wnt, TGF-β, PI3k/Ak, Ras/MAPK, Rho/Rac, and others 19-21. It is believed that a greater understanding of the underlying molecular mechanisms of EMT in GC can help identify novel therapeutic targets.

We found that NKCC1 was overexpressed in GC tissues. It promoted EMT in GC cells, and upregulated MMP2 and MMP9 through the MAPK-JNK signaling pathway. NKCC1 is a potential treatment target for GC.

## Materials and methods

### Patients and sample collection

The study was approved by the Ethics Committee of the First Affiliated Hospital of Guangxi Medical University. All the patients were diagnosed with gastric cancer by electronic gastroscope biopsy, excluding other systematic tumors or tumor metastasis to the stomach, and the patients did not receive chemotherapy or radiotherapy before operation. All primary tumor and paired normal gastric epithelial tissues were obtained from 95 pathologically confirmed GC patients who underwent surgical resection at our center between May 2010 and January 2019. The tissue samples were flash frozen and stored at -80°C for further analysis. Informed consent was acquired from all patients.

### Database information acquisition

The Cancer Genome Atlas project database was analysis by GEPIA2 software (http://gepia.cancer-pku.cn/) to determine expression levels of NKCC1 in GC. The RNA-seq expression data of 408 gastric cancer tissue specimens and 211 normal gastric tissues in the database (Log FC > 1, *p*-value < 0.01) were also analyzed.

### Cell culture

Human GC cell lines (HGC-27, BGC-823, SGC-7901, MKN-28, AGS, and MGC-803) were obtained from the Cell Bank of the Chinese Academy of Sciences (Shanghai, China). Normal gastric epithelial cell GES-1 was obtained from Shanghai Fu Xiang Biotechnology Co. Ltd. The GES-1 and AGS cells were proliferated in DMEM (Gibco-BRI, USA) supplemented with 10% FBS (Gibco-BRI, USA), while the others cells were grown in complete RPMI1640 medium. All cells were cultured under 5% CO_2_ at 37°C.

### qRT-PCR

Total RNA was extracted using the NucleoZOL RNA Isolation Kit (Genecompany, Germany) and reverse transcribed using the PrimerScript RT Kit (Takara, China) as per the manufacturers' instructions. The relative amounts of NKCC1 and GAPDH (internal control) mRNAs were measured by qRT-PCR in the ABI (7500) RT-PCR System (Applied Biosystems, USA) using the SYBR Green QRT-PCR Kit (Takara, China). The primers were used: NKCC1 forward 5′-TAAAGGAGTCGTGAAGTTTGGC-3′ and reverse 5′-CTTGACCCACAATCCATGACA-3′; GAPDH forward 5′-TGACTTCAACAGCGACACCCA-3′ and reverse 5′-CACCCTGTTGCTGTAGCCA AA-3′. All samples were tested in triplicate.

### Western blotting

Proteins were extracted from cells and tissues using RIPA lysis buffer (Thermo Fisher Scientific) supplemented with protease inhibitors (Solarbio) and determined using the Bradford protein assay kit (Beyotime). Cell lysates containing equal amount of proteins (30 µg) were separated via SDS/PAGE gels and transferred to 0.22 µm PVDF membranes (Merck Millipore Ltd). Blots were blocked with 5% milk for 30min at 37 °C, and incubated with primary antibodies, including E-cadherin (CST. No, 3195S, 1:1000), Vimentin (CST. No, 5741S, 1:1000), snail (CST, no, 3879S, 1:1000), MMP2 (CST. No, 40994S, 1:1000), MMP9 (CST. no, 13667S, 1:1000), NKCC1 (Proteintech, no,13884-1-AP 1:10000), JNK (Santa Cruz, no, 7345, 1:200), p-JNK (Santa Cruz, no, 6254, 1:200), ERK (ABclonal, no, A19630, 1:1000), p-ERK (ABclonal, no, APO485, 1:1000), P38 (ABclonal, no, A4771, 1:1000), p-P38 (ABclonal, no,AP05261:1000), and GAPDH (Cell Signaling Technology, 1:10000). The blots were probed with secondary antibody (Cell Signaling Technology 1:5000 dilution) for 30min at 37 °C. The positive bands were detected by ECL kit (Pierce).

### Constructs and vectors

NKCC1-shRNA (sh1: 5'-CCAGCACTACTATTATGATAC-3', sh2: 5'-GGTGATTTCGTCATAGGAACA-3', sh3: 5'-CCAATTATCTCAAACTTCTTC-3' shNC: 5'-ACAGAAGCGATTGTTGATC-3') and expression plasmid were synthesized by Genecopoeia, Inc. (Guangzhou, China). The GC cells were transfected with suitable constructs using Lipofectamine 3000 (Thermo Fisher Scientific, USA) as per the manufacturer's instructions. After culturing for 48 h, stable transfectants were screened using polybrene, and the expression levels of target protein and mRNA were analyzed.

### Cell proliferation assay

CCK-8 assay (CCK-8 SAB biotech. College Park, MD, USA) was used for cell proliferation detection. Following the manufacturer's protocol. The Cells were seeded into 6-well plates at a density of 1.0×10^5^ cells per well and incubated for 24 h in medium supplemented with 5% FBS, and incubated at 37 °C with 5% CO2. At 24 h after transfection, cells were digested with trypsin and seeded in triplicate into 96-well plates (3×10^4^ cells/ well). Each well was incubated with 10 μl/well of Cell Counting Kit-8 solution for 2 h daily for 5 days. Optical density at 450 nm was measured on a microplate reader. Three independent experiments were performed.

### Immunofluorescence

Cells were fixed with 4% polymerized formaldehyde (Solarbio) for 15 min and then permeabilized with 0.1% Triton-X100 (Solarbio) for another 15 min. The samples were incubated overnight with anti-E-cadherin and anti-vimentin antibodies (Protein Tech, 1:50) at 4 °C. After incubating with the secondary antibody (1:200) in 5% BSA (Solarbio) for 1 h at 37 °C, the cells were counterstained with DAPI for 25 min. The cells were observed using a fluorescence microscope and photographed (200× magnification).

### Wound-healing assay

Cells (5×10^5^) were seeded in a 6-well plates and cultured cells reached 85% confluence. The monolayer was scratched longitudinally across the plates with a sterile p200 pipette tip to simulate a wound. The cellular debris was removed by PBS. Cell migration was observed 0, 12, and 24 h after wounding.

### Cell invasion and migration assays

The suitably transfected GC cells were plated in the top chambers of Corning Incorporated Cell Culture Inserts (#3422; Franklin Lakes, New Jersey, USA) or Corning Incorporated BioCoat™ Matrigel™-coated Invasion Chamber (#3422) in 2% FBS, and the lower chambers were placed with media containing 5% FBS as the chemoattractant. The cells were incubated at 37 °C in a humidified atmosphere containing 5% CO_2_ for 24 h or 48h. The migrated cancer cell on the lower surface were fixed with 95% alcohol, and stained using 0.1% crystal violet (Solarbio). The number of invaded cells was counted and photographed (100× magnification) under a phase contrast microscope (four random fields per well).

### Statistical analysis

Experiments data are summarized as mean ± SD of three times repeation. GraphPad Prism 5 (GraphPad Software, Inc, USA) was used to statistical analysis. The differences between groups were compared using two-tail Student's *t* test and variance. *P* values were less than 0.05 were considered statistically significant.

## Results

### NKCC1 is overexpressed in primary GC tissues

**Figure 1:**
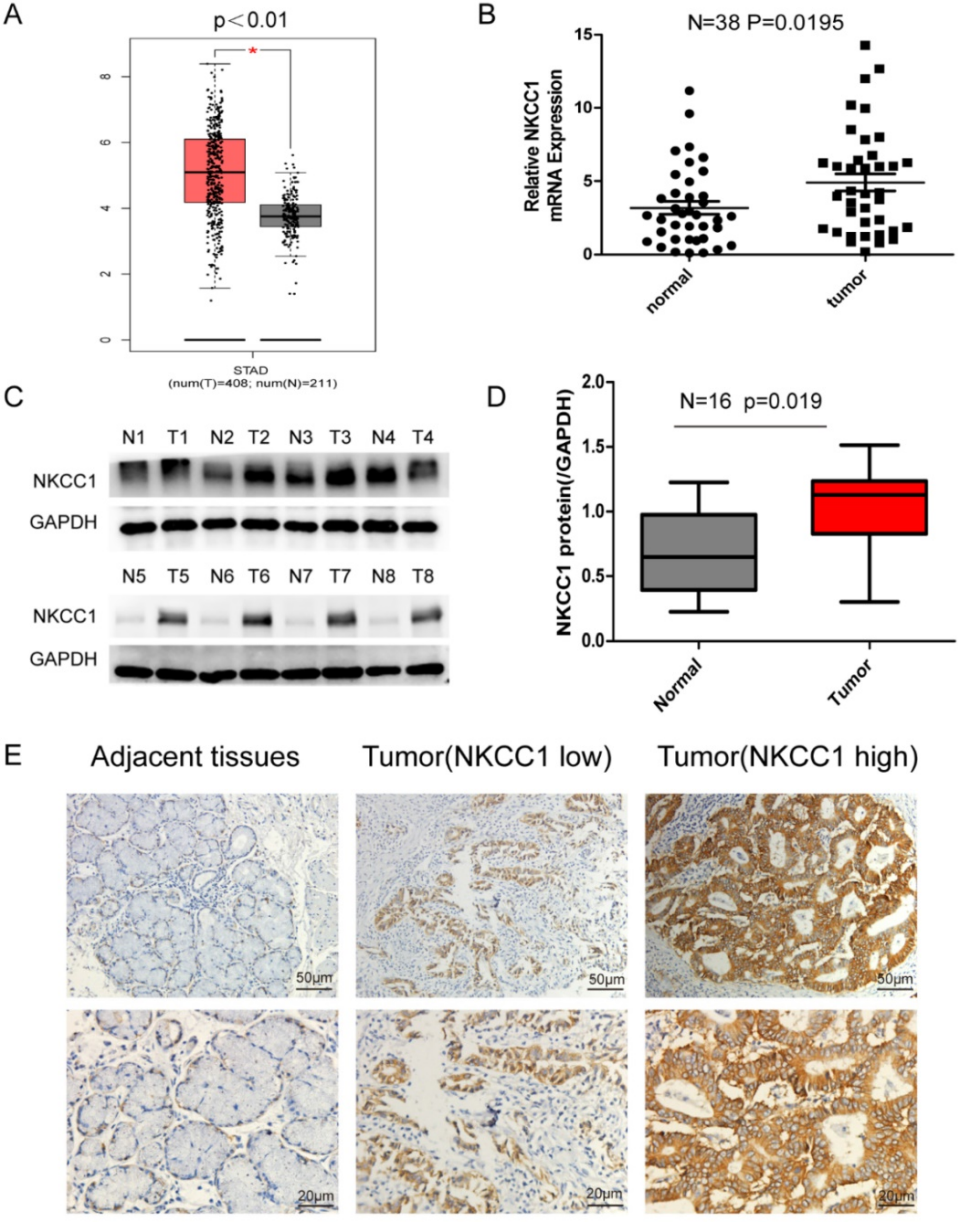
** Expression of NKCC1 in Gastric cancer tissues and normal Gastric tissues. A.** NKCC1 mRNA levels in 408 GC tissues and 211 para-tumor tissues in TCGA database. **B.** NKCC1 mRNA levels in 38 GC tissues and paired adjacent tissues. **C-D.** Western blotting showing NKCC1 protein expression levels in GC tissues and para-tumor tissues with GAPDH as the internal reference control and the quantification of the three independent repeated experiment were shown (n=16, p=0.019). **E.** Representative immunohistochemistry images showing *in situ* NKCC1 expression in the tumor, magnification - 200X, Scale bars: 50 µm, magnification - 400X, Scale bars: 20 µm.

NKCC1 was upregulated in the 408 GC tissues compared to the 211 normal gastric epithelial tissues (*P* < 0.01; Figure 1A) in TCGA datasets as per GEPIA (http://gepia.cancer-pku.cn/). Consistent with this, NKCC1 mRNA levels were significantly higher in the gastric tumors relative to the paired adjacent normal tissues in our cohort (Fig. 1B), and 87.5% of the GC tissues overexpressed NKCC1 protein compared to the paired adjacent normal samples (Fig. 1C, D). Representative immunohistochemistry images showed *in situ* NKCC1 expression in the tumor (Fig. 1E). As shown in Table 1, the clinical relevance of high NKCC1 expression was significantly associated with GC Tumor size (*P* = 0.039), TNM stage (*P* = 0.034) and lymphatic node metastasis (*P* = 0.035) but did not show any significant correlation to age, gender, tumor localization and differentiation. Altogether, NKCC1 is significantly upregulated in GC tissues and can be a potential oncogenic factor.

### Knockdown of NKCC1 inhibited the proliferation, migration and invasion of GC cell lines

**Figure 2:**
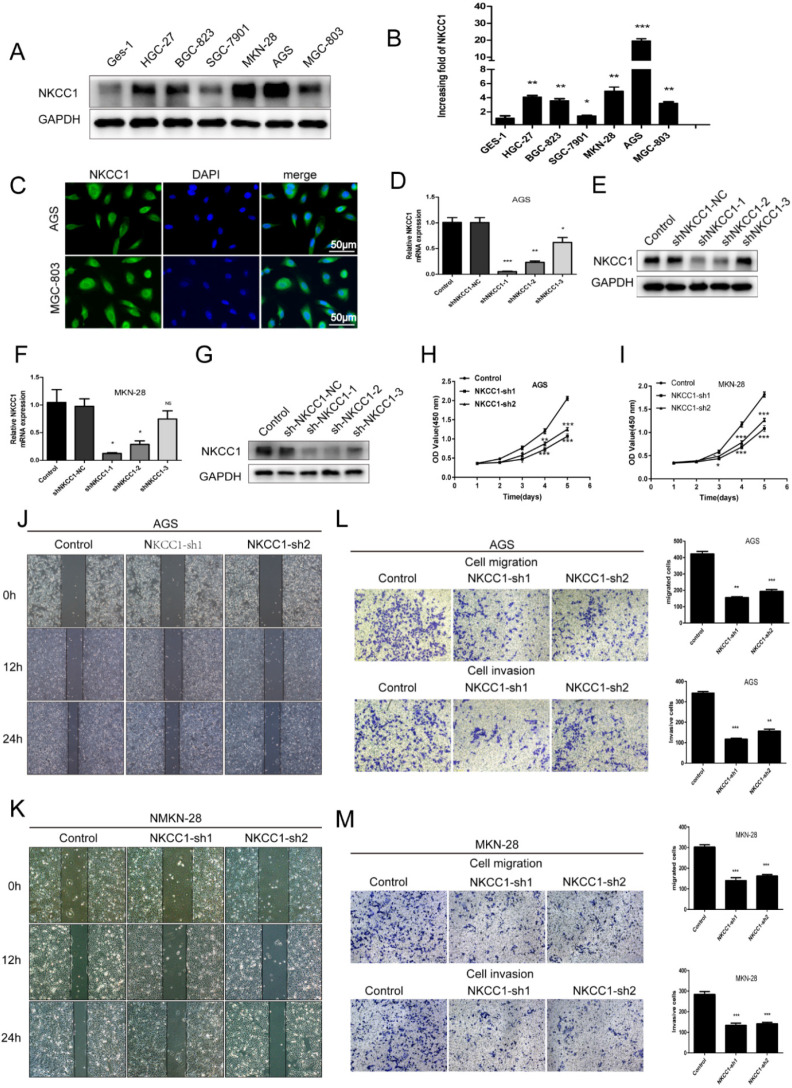
** Knockdown of NKCC1 inhibited the proliferation, migration and invasion of GC cells. A-B.** NKCC1 protein and mRNA levels were observed in the GC cell lines and GES-1 cells. **C.** Representative immunofluorescence images showing localization of NKCC1 on the plasma membrane of the GC cells (magnification - 200X, Scale bars: 50 µm). **D-G.** NKCC1 mRNA and protein levels in GC cells transfected with shNKCC1 and scrambled control. **H-I.** The proliferation capacities were detected by CCK8 assays in GC cells transfected with shNKCC1 and scrambled control. **J-K.** Representative images of wound healing assay showing the *in vitro* migration of control and shNKCC1 GC cells (magnification - 40X, Scale bars: 200 µm). **L-M.** Transwell assay showing *in vitro* migration and invasion of control and shNKCC1 GC cells (magnification - 100X, Scale bars: 100 µm). * *P* < 0.05; ** *P* < 0.01; *** *P* < 0.001.

To elucidate the potential biological function of NKCC1 in GC development, the expression of NKCC1 in GC cell lines (HGC-27, BGC-823, SGC-7901, MGC-803, AGS, MKN-28) was first evaluated by Western-blot and qRT-PCR. NKCC1 protein and mRNA were markedly upregulated in the GC cells compared with GES-1 cells (Fig. 2A, B). In addition, NKCC1 was mainly localized in the vesicles and plasma membrane in AGS and MGC-803 cells (Fig. 2C). This is particularly important because the exact function of NKCC1 varies depending on its intra- or extracellular localization. To further demonstrate the role of NKCC1 in GC progression, NKCC1 was knocked down in AGS and MKN-28 lines (Fig. 2D-G). As Figure 2H and I illustrate, stable knockdown of NKCC1 obviously suppressed growth of GC cells *in vitro* in the CCK-8 assays. As Figure 2J and K illustrate, stable knockdown of NKCC1 decreased migration of GC cells in the wound healing assay. Furthermore, the transwell assay showed an obvious reduction in the motility and invasiveness of the NKCC1-knockdown GC cells compared to the control cells (Fig. 2L M). These results implied that knocking down NKCC1 inhibited the proliferation, invasiveness and metastasis of GC cells.

### Overexpression of NKCC1 enhances the proliferation, migration and invasion of GC cell lines

**Figure 3:**
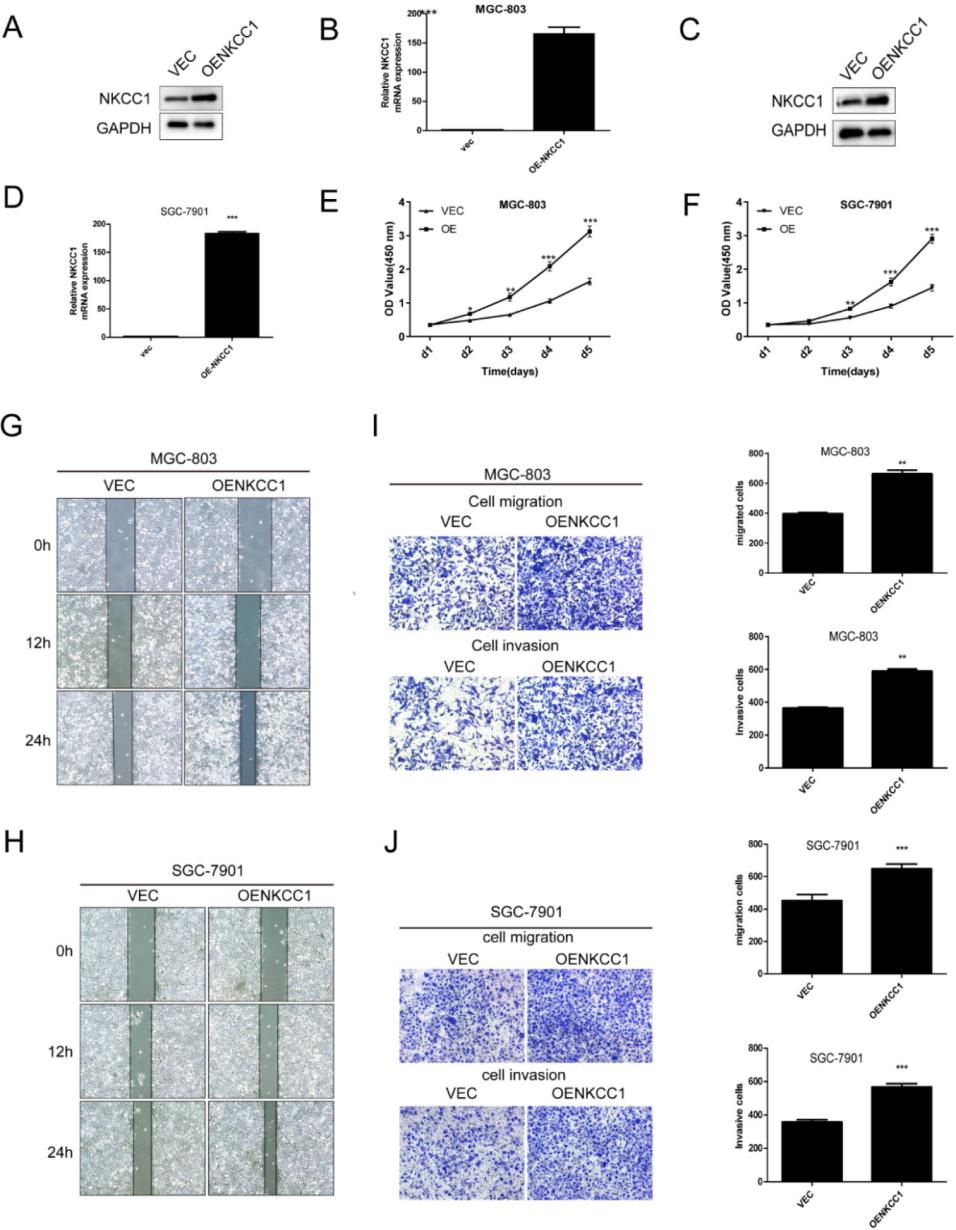
** Overexpress of NKCC1 enhances the proliferation, invasion and migration of GC cells. A-D.** NKCC1 protein and mRNA levels in GC cells transfected with NKCC1 expression plasmid. **E-F.** The proliferation capacities were detected by CCK8 assays in GC cells transfected with the control or the NKCC1-overexpressing plasmid. **G-H.** Wound healing assay showing the *in vitro* migration of control and NKCC1-overexpressing GC cells (magnification - 40X, Scale bars: 200 µm). **I-J.** Transwell assay showing *in vitro* invasion and migration of control and NKCC1-overexpressing GC cells (magnification - 100X, Scale bars: 100 µm). ** *P* < 0.01; *** *P* < 0.001.

NKCC1 was overexpressed in MGC-803 and SGC-7901 cells (Fig. 3A-D). In contrast, stable overexpressed of NKCC1 significantly accelerated cell growth (Fig. E-F). and markedly increased migration of GC cells in the wound healing assay (Fig. 3G, H), and the transwell assay indicated a significant increase in the migration and invasiveness of the overexpressed NKCC1 GC cells compared to the control cells (Fig. 3I, J). Taken together, intracellular NKCC1 enhances the proliferation, invasiveness and metastasis of GC cells.

### NKCC1 induced EMT and increased MMP2/9 expression in GC cell

**Figure 4:**
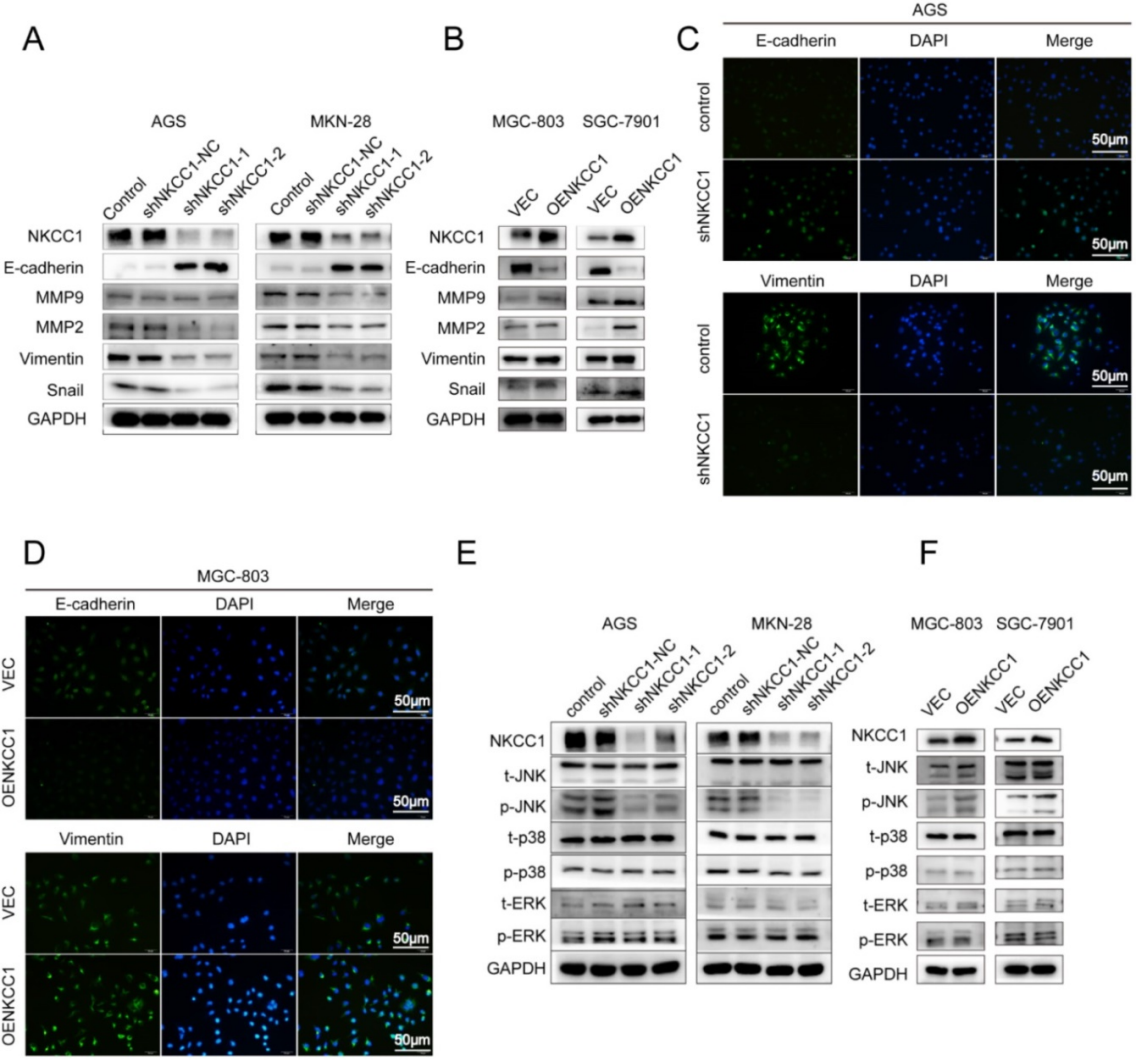
** NKCC1-induced EMT activates the MAPK-JNK pathway in GC cells.** Western blotting showing expression levels of EMT makers in **A.** NKCC1-knockdown GC cells and **B.** NKCC1-overexpressing GC cells. **C-D.** Representative immunofluorescence images showing *in situ* expression of E-cadherin and vimentin in GC cells treated as above (magnification - 200X, Scale bars: 50 µm). **E-F.** Immunoblots showing expression levels of JNK/p-JNK, ERK/p-ERK and p38/p-p38 in E. NKCC1-knockdown GC cells and **F.** NKCC1-overexpressing GC cells.

EMT and extracellular matrix remodeling are critical for cancer cell migration and invasion and are thus key to metastasis 22. Therefore, we analyzed the levels of EMT-related proteins in the NKCC1-knocked down and overexpressing GC cells. As shown in Figure 4A, knocking down NKCC1 downregulated the mesenchymal marker EMT-related transcription factors Snail and Vimentin, and ECM proteins MMP-2 and MMP-9, and upregulated epithelial E-cadherin. In contrast, overexpression of NKCC1 upregulated Vimentin, Snail, MMP-2 and MMP-9 but downregulated E-cadherin (Fig. 4B). Thus, NKCC1 promotes EMT of GC cells.

### NKCC1 induced EMT of GC cells by activating the MAPK-JNK pathway

**Figure 5:**
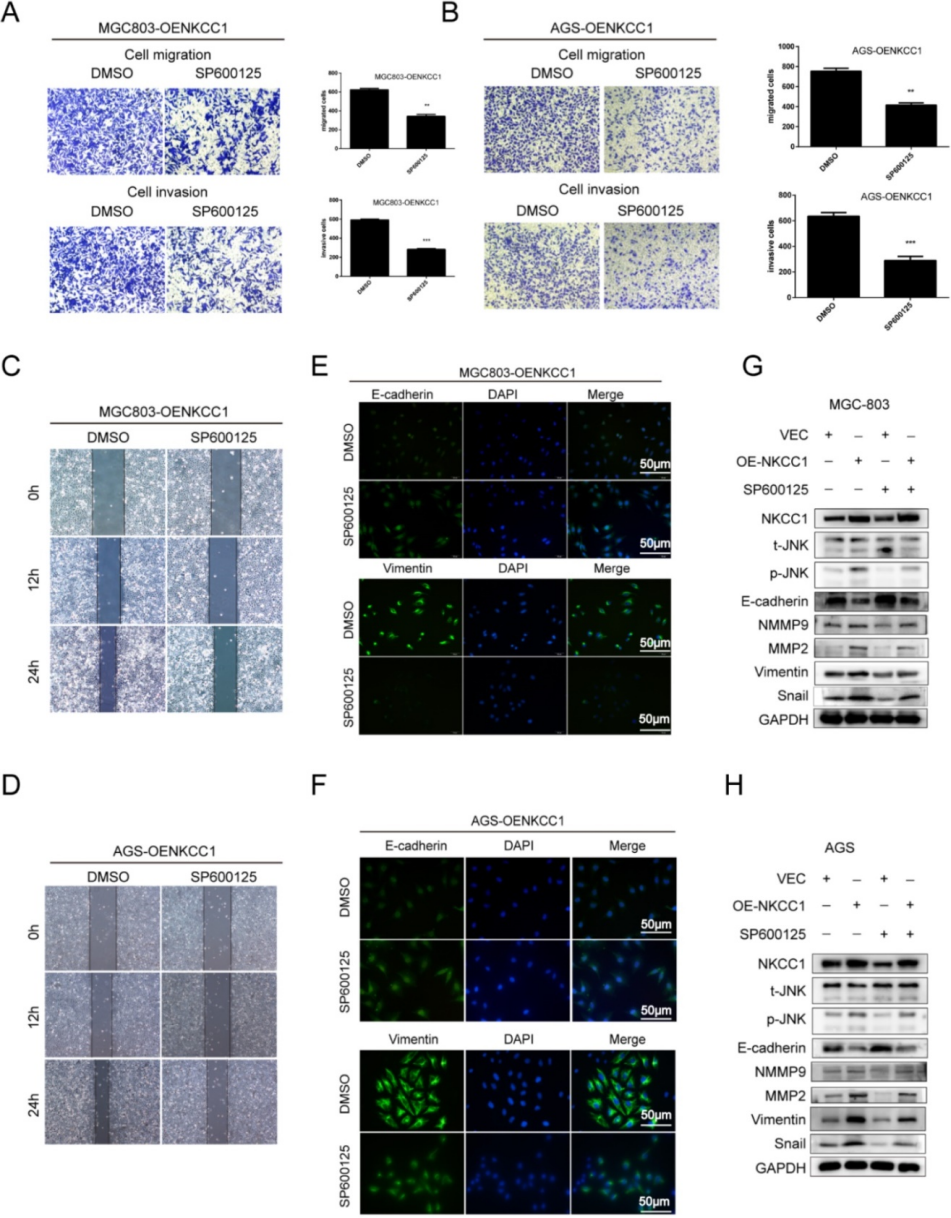
** The MAPK-JNK pathway mediates the pro-metastatic effects of NKCC1 in GC cells. A-B.** Transwell assay showing invasion and migration of MGC-803 and AGS cells treated as above (magnification - 100X, Scale bars: 100 µm). The experiments were repeated 3 times, and the results were summarized as mean ± SD (**, *P* < 0.01). **C-D.** Wound healing assay showing *in vitro* migration of NKCC1-overexpressing MGC-803 and AGS cells treated with JNK inhibitor SP600125 (magnification - 40X, Scale bars: 200 µm). **E-F.** Representative immunofluorescence images showing *in situ* expression of vimentin and E-cadherin in MGC-803 and AGS cells treated as above (magnification - 200X, Scale bars: 50 µm). **G-H.** Immunoblots showing expression levels of p-JNK, ERK, E-cadherin snail, vimentin, MMP2 and MMP9 in MGC-803 and AGS cells treated as above.

The MAPK-JNK/p38 pathway has been implicated in EMT, and the JNK pathway in particular is essential for the progression and maintenance of EMT-related phenotypes and cellular changes 23-24. Therefore, we analyzed the levels of the MAPK-JNK pathway proteins in GC cells following NKCC1 knockdown or overexpression. The p-JNK/JNK ratio was significantly reduced after silencing NKCC1 (Fig. 4E), while no significant change was observed in the phosphorylation of P38 and ERK. As expected, p-JNK/JNK increased markedly in the NKCC1-overexpressing cells whereas p-ERK and p-P38 were not affected (Fig. 4F). To determine whether the MAPK-JNK signaling pathway mediates the metastatic effects of NKCC1, the JNK inhibitor SP600125 (Selleckchem, Inc.) was dissolved in DMSO, we treated the NKCC1-overexpressing MGC-803 and AGS cells with 10 μM SP600125 and found that the invasion and migration of the cells were significantly inhibited (Fig. 5A-D). Immunofluorescent assay also confirmed the changes of EMT markers (vimentin and E-cadherin) after SP600125 treatment in MGC-803 and AGS cells (Fig. 5E-F). Consistent with this, SP600125 downregulated the mesenchymal markers and EMT-related transcription factors in the MGC-803 and AGS cells overexpressing NKCC1 and upregulated E-cadherin (Fig. 5G-H). Overall, NKCC1 regulates EMT by the MAPK- JNK pathway to promote GC invasion and migration.

## Discussion

NKCC1 is an active Na+/K+/ 2Cl- cotransporter, which regulates the changes in cell volume by controlling the intracellular content of water and sodium. Ion transporters and ion channels have recently gained attention for their potential tumorigenic roles. They regulate the intracellular content of water and ions and therefore control cell volume. The CI- channel (CLIC1) enhanced the invasion and migration of gastric and liver cancer cells 25-26, and K+/Ca2+ channels are frequently overexpressed in colon cancer 27-28. Ectopic expression of NKCCI induced the proliferation and phenotypic transformation of mouse fibroblasts 29. In addition, NKCC1 has been reported to play an important role in many different tumorigenesis, it is aberrantly expressed in gastric cancer 9, esophageal cancer 30, meningioma 25, liver cancer 11, and glioma 10 and functions as an oncogene 9-11, 30. We found that NKCC1 was highly expressed in a variety of tumors through analyzing The Cancer Genome Atlas project database using GEPIA software (http://gepia.cancer-pku.cn/). NKCC1 was also highly expressed in GC tissues by analyzing the RNA-seq expression data of 408 GC tissue specimens and 211 normal gastric tissues in the database. The subsequent experiments will be carried out for further verification.

Previous studies show that NKCC1 promotes the invasion and migration of meningioma 31, glioma 10, 32, and liver cancer 11 cells and proliferation of esophageal cancer 30 and liver cancer 11 cells. In addition, a previous study reported that blocking NKCC1 in GC cells can inhibit cell cycle progression by disrupting the intracellular Cl- concentration 33. Significantly higher levels of NKCC1 were detected in GC tissues patients compared to the paired para-tumor tissues, which was closely related to the Tumor size, lymph node metastasis and TNM stage, suggesting that upregulated expression of NKCC1 in GC might facilitate the tumor growth and metastatic phenotype. This was consistent with the data from TGCA datasets, further confirming that NKCC1 is highly expressed in GC tissues. These findings underscore a potentially important role of NKCC1 as an underlying biological mechanism in the progression of GC. To clarify the biological function of NKCC1 in regulating GC cell growth, motility and invasiveness, a series of *in vitro* assays were conducted. The results showed that knocking down NKCC1 in the GC cell lines inhibited EMT and downregulated the related factors, resulting in lower proliferation, invasion and migration *in vitro*. In *in vitro* experiments, the expression of NKCC1 in gastric epithelial cells (GES-1) and six gastric cancer cell lines was first detected, which was relatively high in AGS and MKN-28 cells, but relatively low in SGC-7901 and MGC-803 cells. The expression of proteins in different locations of the cell plays a different role. Immunofluorescence experimental results indicate that NKCC1 is mainly expressed in the cytoplasm and cell membrane of gastric cancer cells as a plasma membrane protein. Next, we changed the expression of NKCC1 in gastric cancer cells. In contrast, NKCC1 overexpression promoted EMT and increased the proliferation, invasion and migration of GC cells. In addition, it is reported that the matrix metalloproteinases (MMPs) enhanced tumor cell motility and invasion by degrading the extracellular matrix 34-35. Silencing NKCC1 decreased MMP 2/9 levels, while its overexpression produced the opposite effects. The above data suggested that NKCC1 may be a new indicator for the growth and metastasis of gastric cancer, and may act as an oncogene in the progression of gastric cancer as well as plays an important role in the proliferation, invasion and migration of GC cells.

EMT plays an important role in the infiltration, invasion, and distant metastasis of various tumors. EMT not only initiates tumor invasion and migration, but is also an indicator of the distant metastasis ability of tumor cells 36-37. Studies show that EMT promotes GC progression via the MAPK-JNK signaling pathway, and activating this pathway induces tumor EMT 38-40. Consistent with this, knockdown of NKCC1 inhibited EMT-related proteins including Snail, vimentin, MMP2 and MMP9 but increased E-cadherin, while NKCC1 overexpression had the opposite effects. Several studies have shown that JNK promoted EMT and enhanced the invasion and migration of GC cells 24, 38-40, which was consistent with our findings. Furthermore, NKCC1 silencing also inhibited JNK activation without affecting P-38 and ERK. Not surprisingly, therefore, targeted inhibition of JNK in the GC cells overexpressing NKCC1 reversed EMT. Immunofluorescence staining demonstrated that E-cadherin expression was up-regulated and vimentin expression was down-regulated after GC cells overexpressing NKCC1 was treated with the JNK inhibitor SP600125. Hence, NKCC1 may regulate the occurrence of EMT through MAPK-JNK signal pathway. After SP600125, a JNK inhibitor was added to gastric cancer cells with overexpressed NKCC1, the EMT-related proteins were changed. The inactivation of MAPK-JNK reversed the overexpression of NKCC1 and induced EMT. The invasion and migration ability of GC cells with overexpressed NKCC1 was inhibited, indicating that NKCC1 could mediate EMT by activating the MAPK-JNK signal pathway. As a result, it was concluded that MAPK-JNK signal pathway played an important role in the progression of GC mediated by NKCC1.

In summary, NKCC1 acts as an oncogene in GC and promotes invasion and migration through activating the MAPK-JNK/EMT pathway. This study proves NKCC1 to be a potential therapeutic target in GC. NKCC1 was highly expressed in human GC samples, and may be a marker for regulating the prognosis of gastric cancer. NKCC1 could regulate the proliferation, invasion and migration of gastric cancer cells *in vitro*. It was further confirmed that NKCC1 could induce EMT to promote the invasion and migration of GC cells through MAPK-JNK pathway. Our study showed that NKCC1 played an important role in the proliferation and metastasis of gastric cancer and may become a candidate marker for clinical diagnosis and treatment of GC.

## Figures and Tables

**Table 1:** Correlation between NKCC1 expression and patients clinicopathological characteristics in 95 GC patients

Parameters	Total case	NKCC1		*P* value
	95	High expression (N=50)	Low expression (N=45)	
**Age (years)**				0.745
≥68	46	25	21	
<68	49	25	24	
**Gender**				0.959
Male	72	38	34	
Female	23	12	11	
**Tumor size**				0.039*
≥6.5m	40	26	14	
<6.5 cm	55	24	31	
**Lymphatic node metastasis**				0.035*
Negative	32	12	20	
Positive	63	38	25	
**Invasion depth (T)**				0.845
T1-2	12	6	6	
T3-4	83	44	39	
**N**				0.810
N1-2	41	21	20	
N3-4	54	29	25	
**M**				1.000
M0	91	48	43	
M1	4	2	2	
**TNM stage**				0.034*
I-II	42	17	25	
III-IV	53	33	20	

NOTE: The values are statistically significant (*** *P* <0.001; ** *P* <0.01; * *P* <0.05); The 8thTNM Classification of Malignant Tumors proposed by the AJCC/UICC. The mean age at diagnosis is 68 years in patients with GC. Samples are divided into two groups based on the mean age. The mean tumor size was 6.5 cm. They were divided into two groups based on the mean tumor size.

## Abbreviations

NKCC1: Na^+^/K^+^/2CI^-^ cotransporter 1
GC: gastric cancer
EMT: Epithelial-Mesenchymal Transition
qRT-PCR: real-time quantitative polymerase chain reaction
